# The Effectiveness of Metagenomic Next-Generation Sequencing in the Diagnosis of Prosthetic Joint Infection: A Systematic Review and Meta-Analysis

**DOI:** 10.3389/fcimb.2022.875822

**Published:** 2022-06-10

**Authors:** Jun Tan, Yang Liu, Sabrina Ehnert, Andreas K. Nüssler, Yang Yu, Jianzhong Xu, Tao Chen

**Affiliations:** ^1^ Department of Orthopedic Surgery, The First Affiliated Hospital of Zhengzhou University, Zhengzhou, China; ^2^ Department of Clinical Sciences, Orthopedics, Faculty of Medicine, Lund University, Lund, Sweden; ^3^ Department of Trauma and Reconstructive Surgery, BG Trauma Center Tübingen, Siegfried Weller Institute for Trauma Research, Eberhard Karls University Tübingen, Tübingen, Germany

**Keywords:** metagenomics, next-generation sequencing, clinical diagnosis and treatment, arthroplasty, infection disease, prosthetic joint infection

## Abstract

**Background:**

A prosthetic joint infection (PJI) is a devastating complication following total joint arthroplasties with poor prognosis. Identifying an accurate and prompt diagnostic method is particularly important for PJI. Recently, the diagnostic value of metagenomic next-generation sequencing (mNGS) in detecting PJI has attracted much attention, while the evidence of its accuracy is quite limited. Thus, this study aimed to evaluate the accuracy of mNGS for the diagnosis of PJI.

**Methods:**

We summarized published studies to identify the potential diagnostic value of mNGS for PJI patients by searching online databases using keywords such as “prosthetic joint infection”, “PJI”, and “metagenomic sequencing”. Ten of 380 studies with 955 patients in total were included. The included studies provided sufficient data for the completion of 2-by-2 tables. We calculated the sensitivity, specificity, and area under the SROC curve (AUC) to evaluate mNGS for PJI diagnosis.

**Results:**

We found that the pooled diagnostic sensitivity and specificity of mNGS for PJI were 0.93 (95% CI, 0.83 to 0.97) and 0.95 (95% CI, 0.92 to 0.97), respectively. Positive and negative likelihood ratios were 18.3 (95% CI, 10.9 to 30.6) and 0.07 (95% CI, 0.03 to 0.18), respectively. The area under the curve was 0.96 (95% CI, 0.93 to 0.97).

**Conclusion:**

Metagenomic next-generation sequencing displays high accuracy in the diagnosis of PJI, especially for culture-negative cases.

## Introduction

Prosthetic joint infection (PJI), noted as a devastating complication of prosthetic joint implantation, accounts for 25% of failed knee arthroplasties and 15% of failed hip arthroplasties (Bozic et al., 2010; Rietbergen et al., 2016). PJI after joint arthroplasty has extreme adverse effects on cost and quality of life. In recent years, with the increasing number of cases, the proportion of its cost in the healthcare budgets is also increasing (Kallala et al., 2018). It is estimated that each episode of prosthetic infection costs the health service over 20, 000 pounds (Vanhegan et al., 2012).

To date, the timely and accurately diagnosis of PJI is still challenging, especially for the identification of pathogenic microorganisms. Although many methods have emerged for establishing the diagnosis, none has been universally accepted (Moojen et al., 2014). Nowadays, traditional blood testing, such as white blood cell (WBC) count, serum erythrocyte sedimentation rate (ESR), and serum C-reactive protein concentration (CRP) are being widely performed for diagnosing PJI in clinics. Nonetheless, these inflammatory markers are nonspecific for PJI, and sometimes they may even be normal in severe cases of joint infections (Nodzo et al., 2015). In addition, routine microbial culture has also been widely used to identify causative organisms in PJI, but it has a significantly high false-negative rate (Rak et al., 2013; Yoon et al., 2017). It has been reported that approximately 40% of culture-negative cases meet the clinical diagnostic criteria for PJI, which might be due to the restricted growth conditions of specific pathogens and the widespread use of antibiotics (Tande and Patel, 2014). In recent years, matrix-assisted laser desorption ionization time-of-flight mass spectrometry (MALDI-TOF MS) has emerged for the identification of bacterial in clinical laboratories (Peel et al., 2015). The MALDI-TOF MS process is rapid, sensitive and economical in terms of labor and costs involved, in which microbes are identified using either intact cells or cell extracts. Although it shows high accuracy for the direct identification of Gram-negative bacteria from blood culture, the accuracy for Gram-positive bacteria is moderate (Ruiz-Aragón et al., 2018). Therefore, the research and development of a reliable technique for the diagnosis of PJI are urgent for both patients and clinicians.

In recent years, researches on PJI diagnosis have switched to mNGS. mNGS is a rapidly developing technology in terms of both pathogenic microorganism detection and data analysis. It has been shown to play important roles in the diagnosis of cancers, genetic diseases, and infectious diseases (Kwon et al., 2019; Wilson et al., 2019). Compared to PCR, mNGS does not have limitations on the detection of specific pathogens, and it can detect almost all pathogens, such as bacteria, fungi, viruses, and parasites. Furthermore, it allows thousands or even billions of DNA fragments to be sequenced independently at the same time, and its consequence is confirmed through comparison with a dedicated pathogen database (Schlaberg et al., 2017; Gu et al., 2019).

mNGS has shown high value in the diagnosis of pathogens of many infectious diseases. In a study on tuberculous meningitis, the diagnostic sensitivity of mNGS based on cerebrospinal fluid was 84.44%, which was much higher than the 22.2% of traditional cerebrospinal fluid culture (Yan et al., 2020). Another study showed that the sensitivity of mNGS was much higher than that of traditional culture in the pathogen diagnosis of mixed lung infections (97.2% vs 13.9%, *P*<0.01) (Wang et al., 2019).

In 2019, a systematic review discussed the sequencing assays for the diagnosis of PJI and showed a low statistical power, owing to a few studies regarding mNGS was involved in this study. Additionally, this review failed to analyze and evaluate the accuracy and diagnostic value of mNGS for PJI (Li et al., 2019). Herein, we incorporated the latest clinical trials for this systematic review to summarize published studies about mNGS. We also performed a meta-analysis to investigate its diagnostic accuracy for PJI.

## Materials and Methods

The protocol for this review was registered with the PROSPERO database, registration number CRD42020193251. We strictly adhered to the standards of the Preferred Reporting Items for Systematic Reviews and Meta-Analyses (PRISMA) in reporting the findings of this review (Moher et al., 2015).

### Search Strategy

We carefully searched for longitudinal studies (prospective or retrospective case-control, prospective cohort, retrospective cohort, case-cohort, nested-case control trials) reporting on the use of mNGS for PJI in MEDLINE, EMBASE, China National Knowledge Internet (CNKI), and Cochrane Library databases from inception to July 2021. A systematic literature search was performed to obtain all of the published articles focusing on mNGS diagnosis of PJI. Vocabulary and syntax were adjusted according to the different databases. We mainly used “prosthetic joint infection”, “periprosthetic joint infection”, “PJI”, “prosthesis-related infections”, “prosthesis infection”, “infection”, and “metagenomic sequencing”, “mNGS”, “metagenomic next-generation sequencing”, “shotgun metagenomics”, “genomics”, “genetic diagnosis”, “sequencing”, as the search target keywords. The exact retrieval strategy is demonstrated in **Supplementary S1**. Reference lists of the retrieved articles were manually scanned for all relevant additional studies and review articles.

### Study Selection

The screening was performed in two stages, title and abstract screening, followed by full-text screening. A gold standard for diagnosing PJI has not been established, and different studies may adopt different reference standards. Among these reference standards, Musculoskeletal Infection Society (MSIS) (Parvizi et al., 2011) and Infectious Diseases Society of America (IDSA) (Osmon et al., 2013) are commonly used. We included studies with different reference standards, and then investigated the heterogeneity between MSIS and IDSA as reference standards through subgroup analysis. Two researchers independently reviewed the title and abstract of each study to select those likely to meet the inclusion criteria. In the initial stage of the screening, 10 to 12 articles were used to confirm the agreement between the researchers. To achieve at a consensus, any discrepancy was resolved by discussion or with the assistance of a third reviewer. After full-text screening, a list of excluded studies with reasons for exclusion was performed.

Studies were considered eligible for inclusion if they met the following criteria: (1) patients with suspected PJI following primary or revision total hip or knee arthroplasty; (2) focus on mNGS-based diagnosis of PJI; (3) the diagnosis of PJI was confirmed by MSIS or IDSA; (4) false positive (FP), true positive (TP), false negative (FN), and true negative (TN) were provided to construct the 2 × 2 contingency table. Articles were excluded based on the following criteria: (1) Irrelevant reviews, letters, personal opinions, book chapters, and meeting abstracts; (2) insufficient data to calculate sensitivity and specificity; (3) mNGS and PJI were not studied.

### Quality Assessment

The quality of the included studies was evaluated by two researchers using the revised Quality Assessment of Diagnostic Accuracy Studies (QUADAS)-2 (Whiting et al., 2011), which is comprised of four key domains that focus on patient selection, index test, reference standard, flow, and timing. Signaling questions were applied to assess the risk of bias and clinical applicability. The overall risk of bias and applicability was summarized as low, high, or unclear.

### Data Extraction

Two reviewers independently extracted the data from the included studies using a standardized form. Data extraction included the following items: last name of the first author; publication year; study population and regions; false and true positives and negatives; sample site and type; reference standard and study design. To deal with absent or unclear data, we tried to contact the study authors.

### Statistical Analysis

Overall pooled sensitivity, specificity, positive likelihood ratio (PLR), negative likelihood ratio (NLR), diagnostic odds ratio (DOR), and the corresponding 95% CI for the diagnosis of PJI were calculated using a bivariate meta-analysis framework. We also tested the pooled diagnostic value of mNGS through the summary receiver operating characteristic (SROC) curve and the area under the SROC curve (AUC). We assessed heterogeneity among the studies using the chi‐squared and I2 tests. Moreover, subgroup and sensitivity analyses were undertaken to explore the potential sources of heterogeneity. All analyses were undertaken by using RevMan 5.4 (The Nordic Cochrane Centre, The Cochrane Collaboration, London, UK 2020) and Stata 15.0 (Stata Corporation, College Station, TX, USA), and a value of *P* < 0.05 was considered statistically significant.

## Results

**Figure 1:**
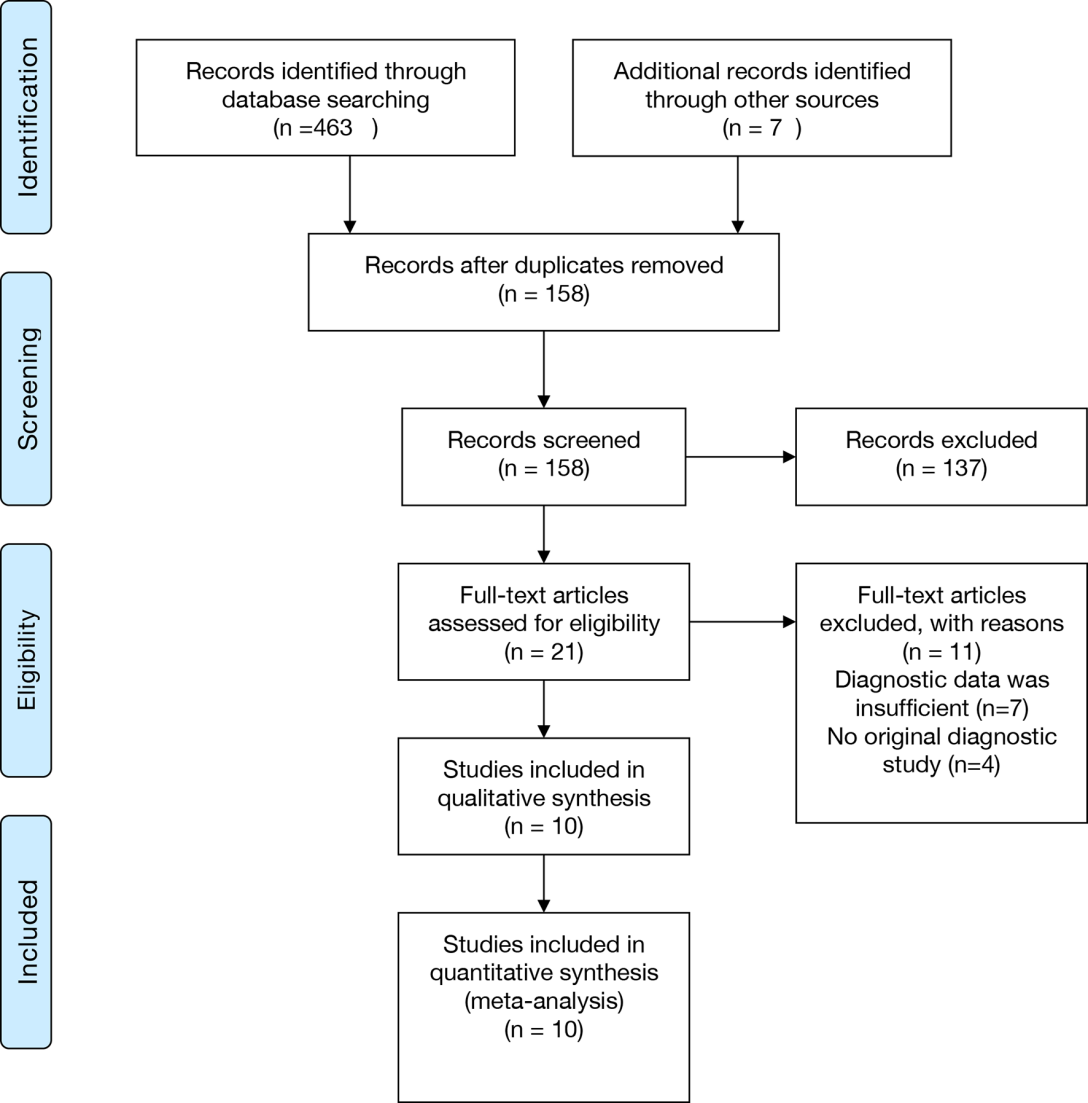
PRISMA flowchart. PRISMA, Preferred Reporting Items for Systematic Reviews and Meta-Analyses.

The selection process was shown in the Preferred Reporting Items for Systematic Reviews and Meta-Analyses (PRISMA) flowchart in **Figure 1**. 380 relevant articles were identified for initial review by systematically searching in the aforementioned databases. Of the identified 380 articles, 253 duplicates were excluded. Then, 106 articles were excluded due to inappropriate article types (reviews, comments, or letters). After reading the remaining 21 articles in full text, seven were excluded due to insufficient data, and four were excluded due to not being an original diagnostic study. Ten studies were finally included in this meta-analysis (Ivy et al., 2018; Thoendel et al., 2018; Huang et al., 2019; Zhang et al., 2019; Cai et al., 2020; Fang et al., 2020; Huang et al., 2020; Wang et al., 2020; He et al., 2021; Yu et al., 2021).

**Figure 2:**
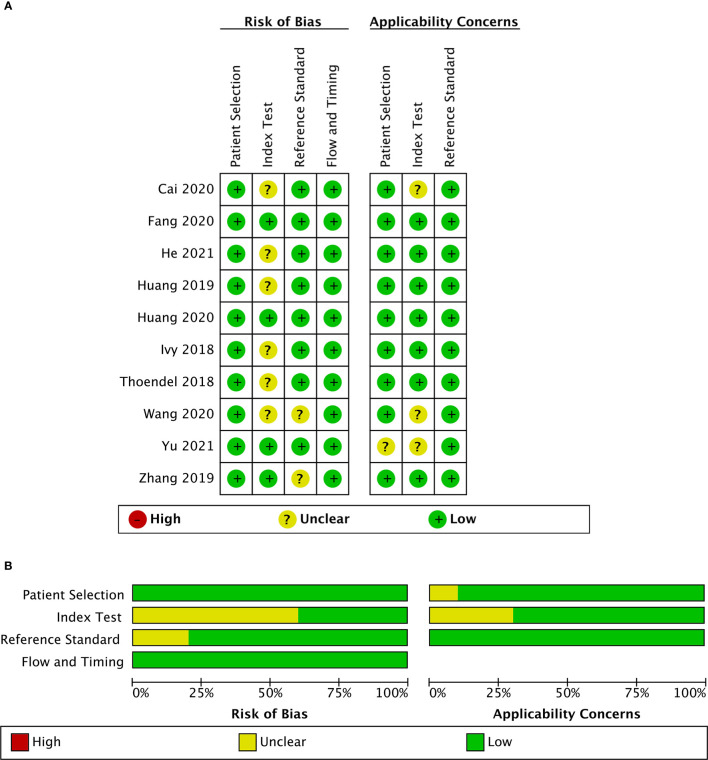
Risk of bias and applicability concerns summary **(A)**; risk of bias and applicability concerns graph **(B)**.

These 10 studies, including a total of 955 patients, were published between 2018 and 2021. Among the included studies, two (Huang et al., 2019; Yu et al., 2021) were conducted retrospectively, and the other studies were conducted prospectively. Eight studies (Ivy et al., 2018; Huang et al., 2019; Cai et al., 2020; Fang et al., 2020; Huang et al., 2020; Wang et al., 2020; He et al., 2021; Yu et al., 2021) collected synovial fluid samples before any clinical treatment, six studies (Thoendel et al., 2018; Huang et al., 2019; Zhang et al., 2019; Fang et al., 2020; Wang et al., 2020; He et al., 2021) obtained sonication fluid and two studies (Cai et al., 2020; He et al., 2021) selected periprosthetic tissue for mNGS. The MSIS criteria were used in seven studies (Huang et al., 2019; Zhang et al., 2019; Cai et al., 2020; Fang et al., 2020; Huang et al., 2020; He et al., 2021; Yu et al., 2021), and the other three studies (Ivy et al., 2018; Thoendel et al., 2018; Wang et al., 2020) adopted the IDSA as the only reference standard. Among the ten studies analyzed, nine studies (Thoendel et al., 2018; Huang et al., 2019; Zhang et al., 2019; Cai et al., 2020; Fang et al., 2020; Huang et al., 2020; Wang et al., 2020; He et al., 2021; Yu et al., 2021) focused on both hip and knee while one study (Ivy et al., 2018) only enrolled knee arthroplasty (**Table 1**). A graphical summary of the methodological assessment based on the QUADAS-2 quality assessment for the 10 studies is shown in **Figures 2A, B**.

**Table 1:** Characteristics of the studies that were included.

Study	Country	Patients	Study design	Sample site(s)	Reference standard	Sample type	Antibiotics*	TP	FP	FN	TN
Thoendel et al., 2018	USA	408	Prospective	Hip and knee	IDSA	Sonication fluid	Yes	251	7	62	188
Ivy et al., 2018	USA	168	Prospective	Knee	IDSA	Synovial fluid	Yes	72	4	35	57
Zhang et al., 2019	China	37	Prospective	Hip and knee	MSIS	Sonication fluid	Yes	24	1	0	12
Huang et al., 2019	China	35	Retrospective	Hip and knee	MSIS	Synovial and sonication fluid	Yes	20	1	0	14
Cai et al., 2020	China	44	Prospective	Hip and knee	MSIS	periprosthetic tissue and synovial fluid	No	21	2	1	20
Wang et al., 2020	China	63	Prospective	Hip and knee	IDSA	Synovial and sonication fluid	No	43	1	2	17
Huang et al., 2020	China	70	Prospective	Hip and knee	MSIS	Synovial fluid	Yes	47	1	2	20
Fang et al., 2020	China	38	Prospective	Hip and knee	MSIS	Synovial and sonication fluid	Yes	24	0	1	13
He et al., 2021	China	59	Prospective	Hip and knee	MSIS	Synovial, sonication fluid and tissues	Yes	38	1	2	18
Yu et al., 2021	China	33	Retrospective	Hip and knee	MSIS	Synovial fluid	Yes	13	1	8	11

TP, true positive; FP, false positive; FN, false negative; TN, true negative; MSIS, Musculoskeletal Infection Society; IDSA, Infectious Disease Society of America guidelines.

*Only antibiotics before sampling are considered here.

Included studies were assessed with the QUADAS-2 guidelines, and detailed information is shown in **Supplementary Table S1**. The majority of studies had a low risk of bias for patient selection, reference standard, flow, and timing. For index test bias, six studies were at an unclear risk because the information was insufficient to ensure that the index test results were interpreted without knowledge of the results of the reference standard. Most of the studies in this meta-analysis raised low concerns about applicability.

**Figure 3:**
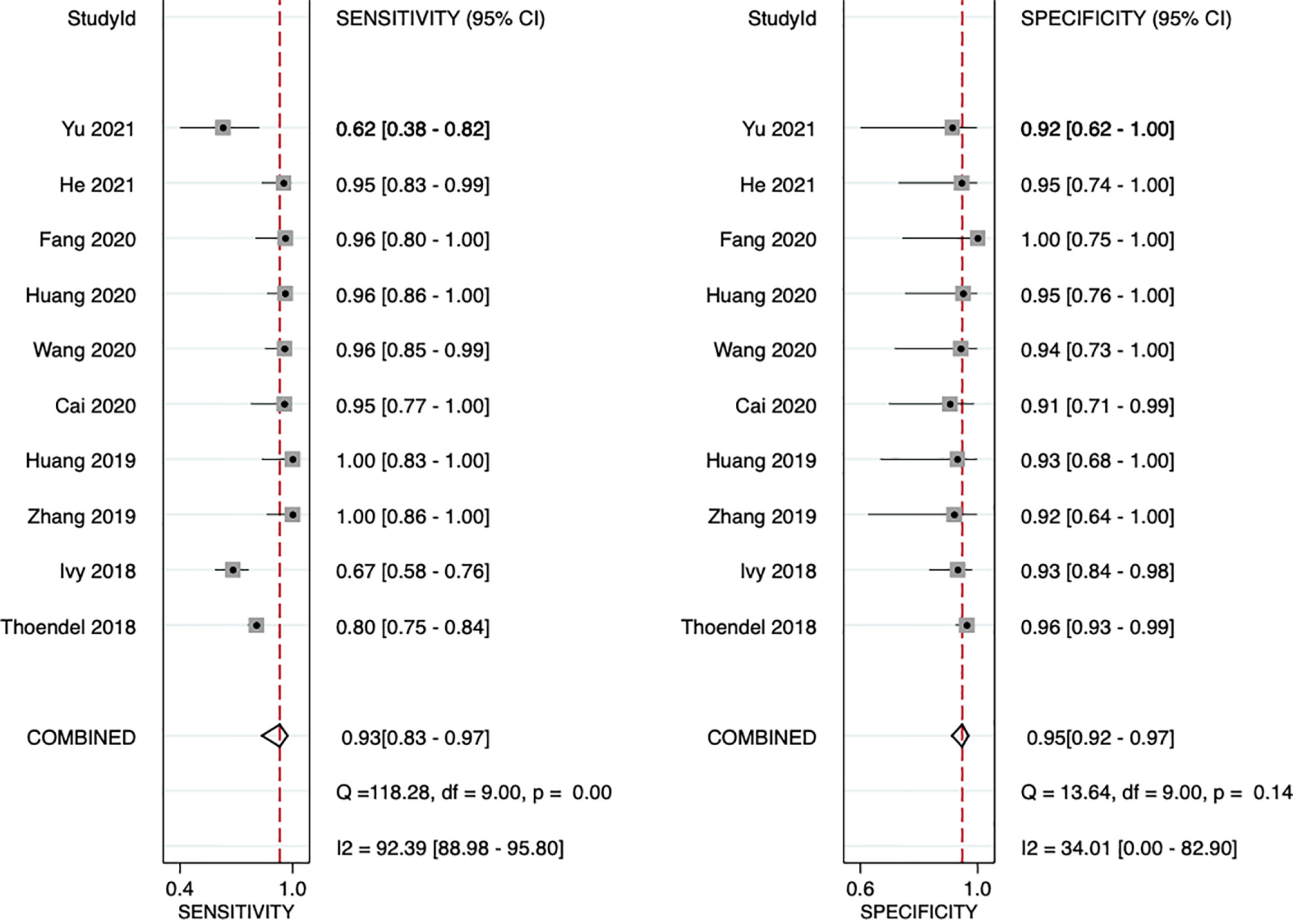
Forest plots for studies on overall mNGS used in the diagnosis of PJI. CI, confidence interval.

**Figure 4:**
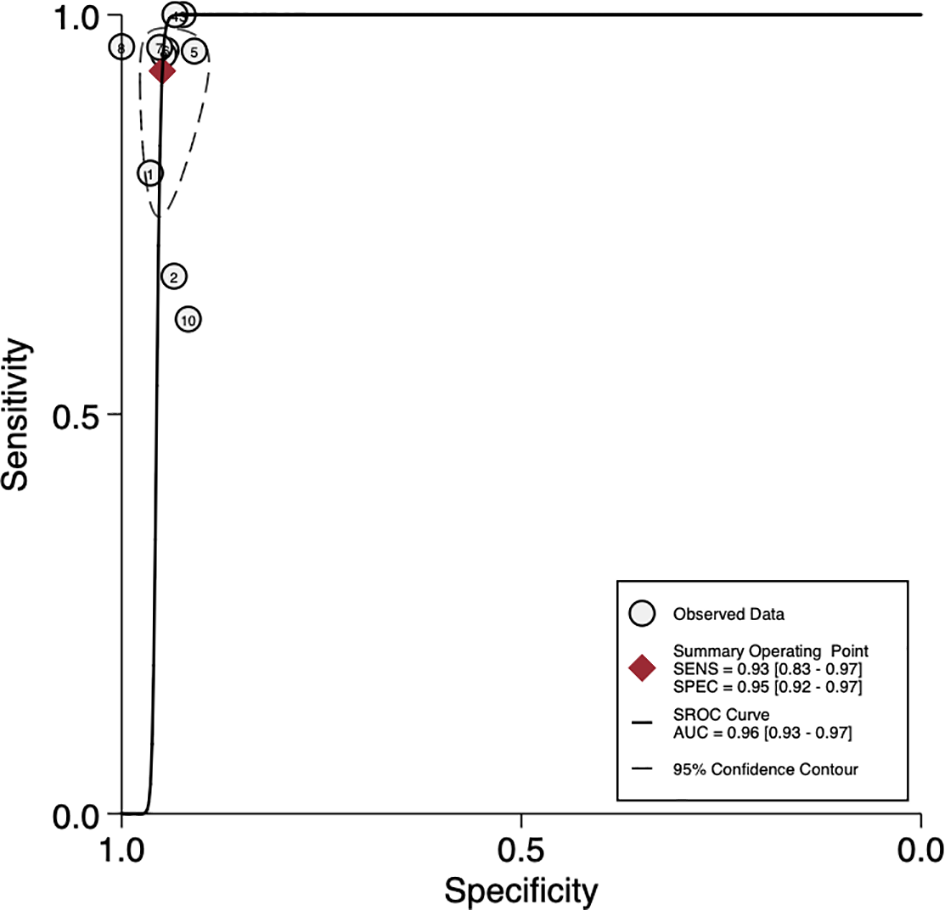
Summary receiver operator characteristic (SROC) curves based on mNGS. AUC, area under the curve; SENS, sensitivity; SPEC, specificity.

The sensitivity and specificity of mNGS for diagnosing PJI are shown in **Figure 3**. The pooled sensitivity was 0.93 (95% CI, 0.83–0.97), specificity was 0.95 (95% CI, 0.92–0.97), positive likelihood ratio was 18.3 (95% CI, 10.9–30.6), negative likelihood ratio was 0.07 (95% CI, 0.03–0.18), and DOR was 247 (95% CI, 84–723). Moreover, we plotted the SROC curve to evaluate diagnostic accuracy (**Figure 4**). AUC was 0.96 (95% CI, 0.93–0.97), suggesting a unique superior diagnostic accuracy of mNGS.

The performance of mNGS in both culture-positive and culture-negative is indicated in **Table 2**. In all 565 specimens tested in all publications, 375 (66.4%) were culture-positive and 190 (33.6%) were culture-negative. In 375 culture-positive specimens, the pathogens identified by culture were also detected by metagenomics in 340 (90.1%) cases. In 190 specimens considered as culture-negative, potential pathogens were detected in 103 (54.2%) using metagenomics.

**Table 2:** Performance of mNGS *versus* culture.

Study	CP-PJI	Organisms identified by metagenomics	CN-PJI	Organisms identified by metagenomics
Cai et al., 2020	16	16 (100%)	6	5 (83.3%)
Wang et al., 2020	35	33 (94.3%)	10	10 (100%)
Huang et al., 2019	13	12 (92.3%)	7	6 (85.7%)
Thoendel et al., 2018	115	109 (94.8%)	98	43 (43.9%)
Ivy et al., 2018	82	69 (84.1%)	25	4 (16.0%)
Huang et al., 2020	39	37 (94.9%)	10	10 (100%)
Zhang et al., 2019	17	17 (100%)	7	7 (100%)
He et al., 2021	34	34 (100%)	6	4 (66.7%)
Fang et al., 2020	18	18 (100%)	6	6 (100%)
Yu et al., 2021	6	5 (83.3%)	15	8 (53.3%)
Total	375	340 (90.1%)	190	103 (54.2%)

Listed are the numbers of samples that were detected by metagenomics in culture-positive and culture-negative PJI samples. CP-PJI, culture-positive prosthetic joint infection. CN-PJI, culture-negative prosthetic joint infection.

**Figure 5:**
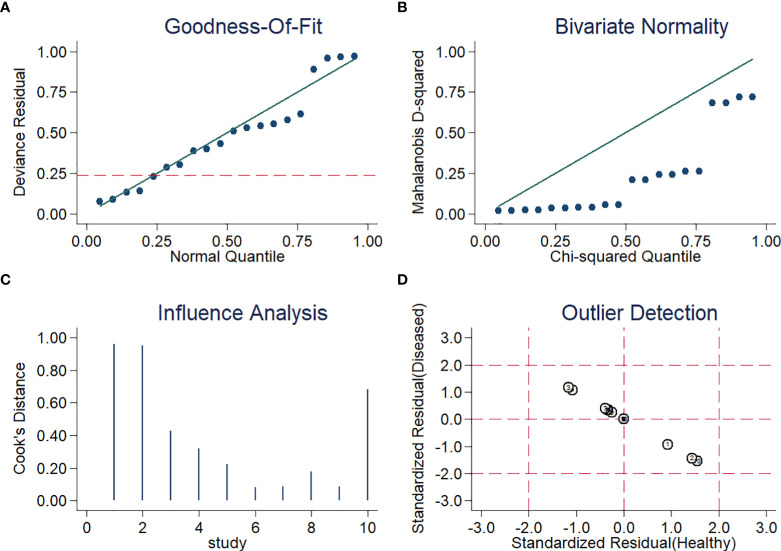
Diagram of **(A)** Goodness-of-fit **(B)** Bivariate normality **(C)** Influence analysis **(D)** Outlier detection.

For sensitivity analysis, the goodness of fit and bivariate normality showed that a random-effects bivariate model is suitable (**Figures 5A, B**). Influence analysis identified that the studies of Thoendel et al. (Thoendel et al., 2018), Ivy et al. (Ivy et al., 2018), and Yu et al. (Yu et al., 2021) were the most dominant studies in weight (**Figure 5C**). Outlier detection implied that the studies of Ivy et al. (Ivy et al., 2018) and Yu et al. (Yu et al., 2021) might be the reason for the heterogeneity (**Figure 5D**). The Spearman correlation coefficient of sensitivity and 1-specificity was 0.418, and the P-value was 0.229, indicating that heterogeneity may not be caused by the threshold effect (**Supplementary S2**). We conducted an univariable meta-regression analysis based on the characteristics of the ten studies to explore the potential sources of heterogeneity. We found that sensitivity was affected by ethnicity, sample site, and study design, while specificity was influenced by ethnicity, sample type, and reference standard (**Figure S1**).

We performed subgroup analysis according to the results of univariate meta-regression to further investigate the sources of heterogeneity. If I^2^ < 50%, or *P* > 0.05, heterogeneity in this subgroup was defined as low. Between these subgroup analyses, ethnicity, sample type, and reference standard showed low heterogeneity (**Table 3**).

**Table 3:** Subgroup analysis of mNGS.

Subgroup	Number of studies	Pooled sensitivity (95% CI)	Pooled specificity (95% CI)	P	I^2^
Ethnicity					
Asians	8	0.94 (0.90-0.96)	0.95 (0.89-0.97)	0.001/0.948	72%/0.0%
Caucasians	2	0.77 (0.73-0.81)	0.96 (0.92-0.98)	0.008/0.341	85.9%/0.0%
Sample type					
Sonication fluid	2	0.816 (0.770-0.856)	0.963 (0.926-0.983)	0.001/0.506	90.2%/0.0%
Synovial fluid	3	0.709 (0.629-0.781)	0.932 (0.857-0.975)	0.001/0.976	86.8%/0.0%
Multiple samples	5	0.961 (0.916-0.985)	0.943 (0.871-0.981)	0.778/0.743	0.0%/0.0%
Reference standard					
IDSA	3	0.787 (0.747-0.823)	0.956 (0.925-0.977)	0.000/0.617	88.5%/0.0%
MISI	7	0.930 (0.886-0.961)	0.939 (0.879-0.975)	0.251/0.902	24.0%/0.0%
Study design					
Prospective	8	0.832 (0.800-0.860)	0.953 (0.926-0.972)	0.00/0.859	86.9%/0.0%
Retrospective	2	0.805 (0.651-0.912)	0.882 (0.636-0.985)	0.00/0.138	92.0%/54.4%

CI, confidence interval; MSIS, Musculoskeletal Infection Society; IDSA, Infectious Disease Society of America guidelines.

Moreover, The Deeks’ funnel plot asymmetry test of pooled DOR with a P-value of 0.20 indicated no significant publication bias (**Figure S2**).

## Discussion

Although mNGS has demonstrated an encouraging value in the diagnosis of pathogens of various infectious diseases, especially for diagnosing tuberculous meningitis and chlamydia psittaci pneumonia, consensus for its clinical application of PJI diagnosis has still not yet been achieved (Chen et al., 2020; Yan et al., 2020). A former meta-analysis suggested that sequencing assays have the potential to improve the clinical diagnosis of PJI, especially for culture-negative cases, but the diagnostic value and accuracy of mNGS in PJI were still unclear (Li et al., 2019). According to our literature search, no previous systematic review or meta-analysis about mNGS in the diagnosis of PJI has been published, which makes it necessary to explore and fill this gap.

Our findings suggested that mNGS had a high accuracy in PJI diagnostics, with a pooled sensitivity of 0.93, a pooled specificity of 0.95, and an AUC of 0.96. The pooled PLR was 18.3, indicating that the probability of an accurate diagnosis of PJI increased by 18.3-fold with positive mNGS testing. Moreover, NLR was 0.07, implying that the probability of a PJI decreased by 93% when the studied mNGS was negative.

Li et al. (Li et al., 2019) showed that the sensitivity, specificity, and AUC of sequencing assays were 0.81, 0.94, and 0.94, respectively. The pooled sensitivity and specificity were both lower than the data of our study (0.81 vs 0.93; 0.94 vs 0.95). The AUC, which is usually used to indicate overall accuracy, was also lower than our study (0.94 *vs*. 0.96), supporting the idea that mNGS might be more effective in the diagnosis of PJI than other sequencing assays. There are several potential reasons for the higher sensitivity and AUC in our study: our study only focused on the diagnostic accuracy of mNGS, while Li et al. used different sequencing methods, including Sanger sequencing, Sequencing by Synthesis and NGS methods. mNGS technology can simultaneously and independently detect pathogens and multiple target genes in the same clinical samples without the need of pre-amplify target sequences (Gu et al., 2019). The ability of mNGS to effectively identify most pathogens in the joint fluid of PJI may have contributed to this result.

In another study of broad-range PCR-based (BR-PCR) diagnosis of PJI (Wang et al., 2020), the pooled sensitivity and specificity were 0.82 and 0.94, respectively, which were both also lower than in our analysis (0.82 vs 0.93; 0.94 vs 0.95). These results were likely caused by different sequencing procedures between mNGS and BR-PCR. The outstanding advantage of mNGS is unbiased sampling, which can broadly identify known and unexpected pathogens and even discover new organisms in an unbiased approach (Gu et al., 2019). BR-PCR is based on the V3-V4 region of 16S rDNA, which can only identify some pathogens at the genus level and may miss the causative pathogens in polymicrobial infection and fungal infections (Dabrowski et al., 2017).

According to subgroup analysis, the effectiveness of mNGS in the diagnosis of PJI among Asians seems to have a better sensitivity than that of Caucasians (0.94 vs. 0.77), while the specificity in Caucasians was slightly higher than in Asians (0.96 vs. 0.95). In fact, the total number of Caucasians studies was much larger than that of Asians (576 vs. 379) and a different platform was used to perform mNGS in the included study. We assume that this may cause the significant difference in sensitivity and specificity between Asians and Caucasians. Therefore, it is necessary to carry out more high-quality clinical trials of different ethnicities to explore racial differences in mNGS. Besides, the significant differences among sample types were considered as the main source of heterogeneity in specificity. Sequencing of sonication fluid seems to have a better specificity than other sample types, while multiple sample types sequencing had better sensitivity than other sample types. In fact, the ultrasonic lysis method can peel the biofilm from the prosthesis surface, increasing the microbial load in the lysate and improving the probability of microbial detection (Huang et al., 2019). Further, compared to the thickened joint fluids that are difficult to centrifuge, ultrasonic lysis fluids could achieve a 20-fold higher concentration of microbial cells after centrifugation and increase the sensitivity of diagnosis. Nevertheless, the ultrasonic lysis procedure may introduce exogenous microbial cells and nucleic acid fragments. Therefore, the additional pathogenic bacteria detected in the ultrasonic lysate should be further verified by specific PCR or other methods to exclude the possibility of exogenous contamination.

Our results showed that the sensitivity of MSIS was better than IDSA (0.930 vs. 0.787), while the specificity of MSIS was lower than IDSA (0.939 vs. 0.956). However, some information important for determining the cases with low virulence levels may be missed by using different reference standards and therefore resulting in the wrong grouping method. For that reason, a common and widely accepted reference standard should be established to help to minimize classification bias.

The main pathogenic microorganisms of PJI obtained by mNGS were Staphylococcus epidermidis (25.1%, 139/553) and Staphylococcus aureus (17.5%, 97/553), which is similar to the common microbiological causes of PJI reported by Tande et al. (Tande and Patel, 2014). It is noteworthy that metagenomics is able to detect most pathogens identified by culture (90.1%) as well as many that were not detected by culture. This occurs particularly in the culture-negative PJI group in which potential pathogens were detected in 54.2% of cases. This result supported the idea that mNGS is a powerful tool to identify PJI pathogens that are difficult to detect in culture-negative infections. Importantly, mNGS will become more accurate and offer more comprehensive microbiologic diagnosis as the technology evolves.

Helping clinical decision-making is the most important value of mNGS. Likelihood ratios and post-test probabilities are useful for clinicians, as they could show the probability that a patient has or does not have PJI, given a negative or positive test result. We also summarized the positive likelihood ratios and negative likelihood ratios to judge the clinical applicability of mNGS for diagnosis (**Figure S3**). PLR >10 and NLR <0.1 represent a high diagnostic accuracy (Wacker et al., 2013). We found that the articles of mNGS from Wang et al. (Wang et al., 2020), Huang et al. (Huang et al., 2020), Fang et al. (Fang et al., 2020), and He et al. (He et al., 2021) had high diagnostic accuracy and clinical applicability. When the pre-test probability was set at 50%, the post-test probability for a positive test result was 95%. When the negative likelihood ratio was set at 0.07, the post-test probability was reduced to 7% for a negative test result (**Figure S4**).

mNGS offers a novel approach to diagnose clinical infectious diseases and address current pitfalls in clinical management. Although the valuable insights of mNGS have already been derived, its use in the diagnosis of PJI is still in its infancy and many challenges still exist (Han et al., 2019). In particular, it is difficult to detect pathogenic virulence and drug sensitivity, which limits its role in guiding the rational selection of antibiotics. Another challenge is no comprehensive and unified background bacteria identification strategy, making interpretation of the sequencing results difficult. It seems inevitable to mix microbial gene sequences during sampling and laboratory testing, which makes it difficult to identify the real pathogen. Moreover, the high cost and lack of timeliness also limit the clinical applications of this technology.

In addition, several limitations of this meta-analysis should be emphasized. It is hard to elucidate whether the sample site had a decisive influence on diagnostic accuracy since the raw data were not provided in the published articles and we cannot divide the data into hip and knee to eliminate heterogeneity. Future studies should focus on the differences in diagnostic accuracy associated with potential sources of heterogeneity, including different arthroplasty sites. Secondly, the gold standard for diagnosing PJI has not been established and we included studies according to different reference standards, which may result in misdiagnosis for PJI (Liu et al., 2018). Thirdly, an antibiotic-free interval before sampling may enhance the ability to detect the causative organism, but through our univariable meta-regression and subgroup analysis, we still could not conclude that antibiotics were the main source of heterogeneity. Finally, studies with positive results are more likely to be published, which can amplify the overall diagnostic accuracy.

## Conclusions

To the best of our knowledge, our study is the first meta−analysis that evaluates the clinical usability of mNGS in the diagnosis of PJI. Our study indicated that mNGS has a superior diagnostic accuracy for PJI and may be particularly useful for culture-negative cases. This systematic review provides effective support for the diagnostic performance of mNGS, which can provide clinicians with recommendations for accurate and effective diagnosis of PJI and antibiotics treatment. Meanwhile, large‐sized and good‐quality studies should be conducted to verify our results and to confirm the clinical value of mNGS in PJI patients.

## Author Contributions

JX and TC were responsible for the idea and concept of the paper. JT and TC built the database. JT and YY analyzed the data. JT wrote the manuscript. YL, SE, and AN critically reviewed and revised the manuscript. All authors contributed to the article and approved the submitted version.

## Conflict of Interest

The authors declare that the research was conducted in the absence of any commercial or financial relationships that could be construed as a potential conflict of interest.

## Publisher’s Note

All claims expressed in this article are solely those of the authors and do not necessarily represent those of their affiliated organizations, or those of the publisher, the editors and the reviewers. Any product that may be evaluated in this article, or claim that may be made by its manufacturer, is not guaranteed or endorsed by the publisher.

## Supplementary Material

Supplementary Figure 1 Univariable meta-regression.

Supplementary Figure 2 The Deeks’ funnel plot of the pooled DOR. DOR, diagnostic odds ratio; ESS, effective sample size.

Supplementary Figure 3 Summary of positive likelihood ratio and negative likelihood ratio for the diagnosis of PJI. LLQ, left lower quadrant; LRN, likelihood ratio negative; LRP, likelihood ratio positive; LUQ, left upper quadrant; RLQ, right lower quadrant; RUQ, right upper quadrant.

Supplementary Figure 4 Fagan nomogram of the mNGS for the diagnosis of PJI.
