# Supplementary figures and images for: The Effectiveness of Metagenomic Next-Generation Sequencing in the Diagnosis of Prosthetic Joint Infection: A Systematic Review and Meta-Analysis
Source: Front Cell Infect Microbiol. 2022 Jun 10;12:875822. doi: 10.3389/fcimb.2022.875822 (PMC9226560; doi:10.3389/fcimb.2022.875822)

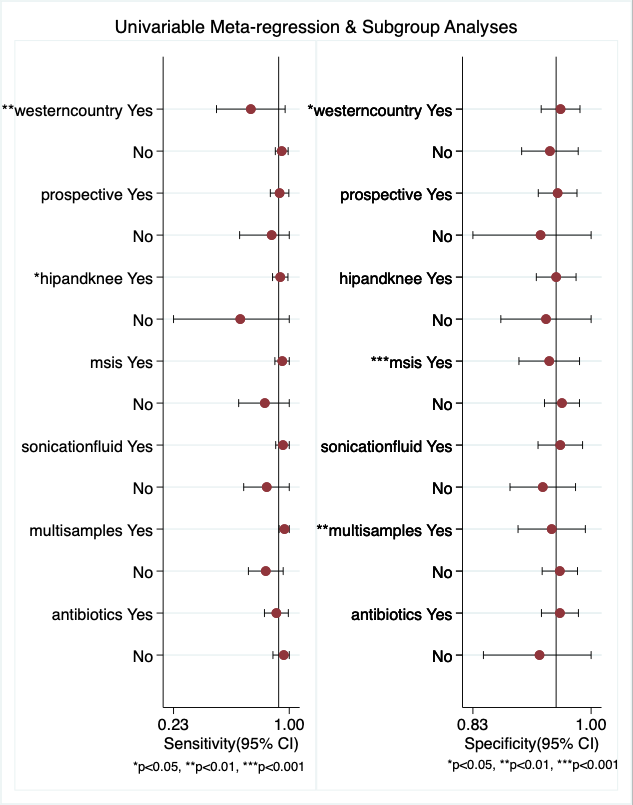

Supplement: Supplementary Figure 1 — Univariable meta-regression. [file Image_1.tif]

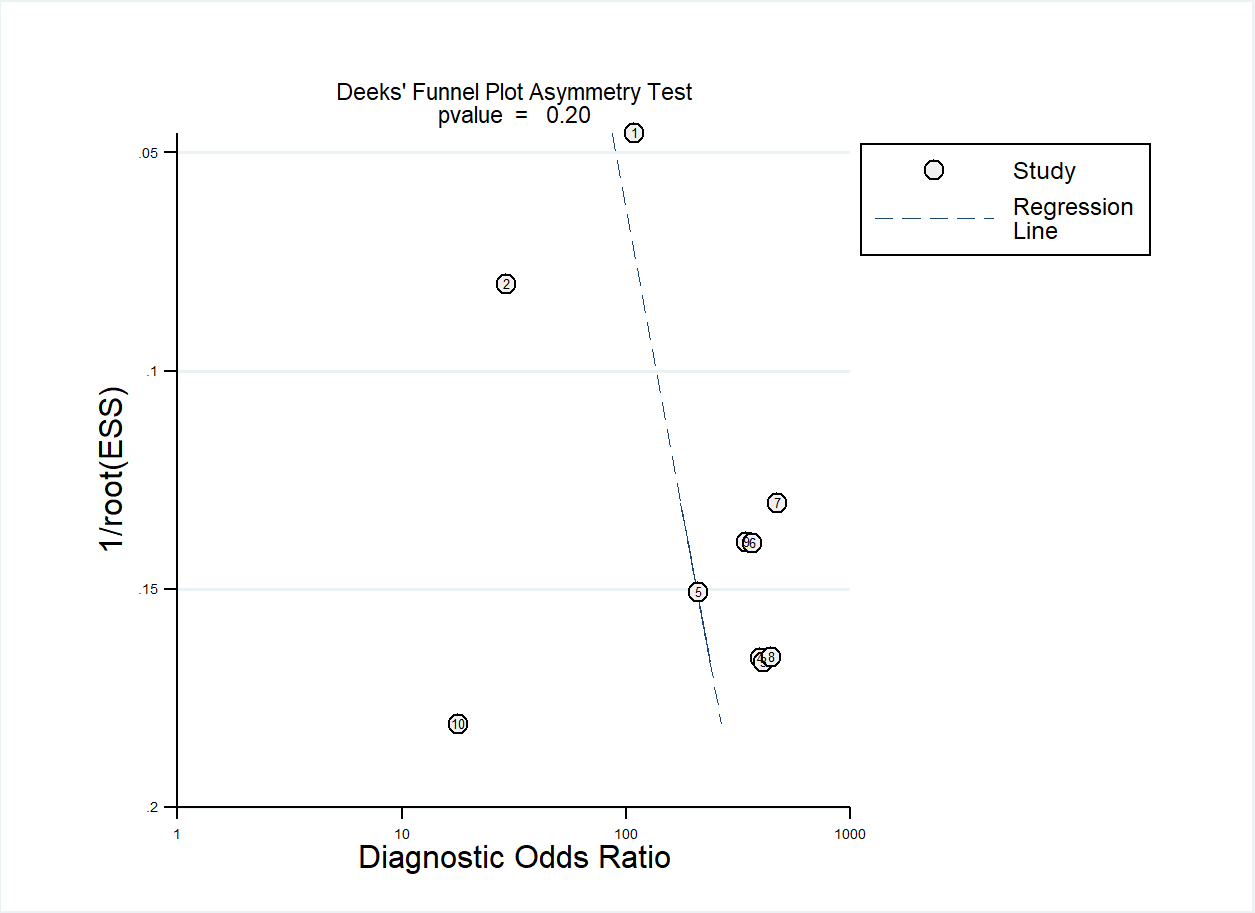

Supplement: Supplementary Figure 2 — The Deeks’ funnel plot of the pooled DOR. DOR, diagnostic odds ratio; ESS, effective sample size. [file Image_2.tiff]

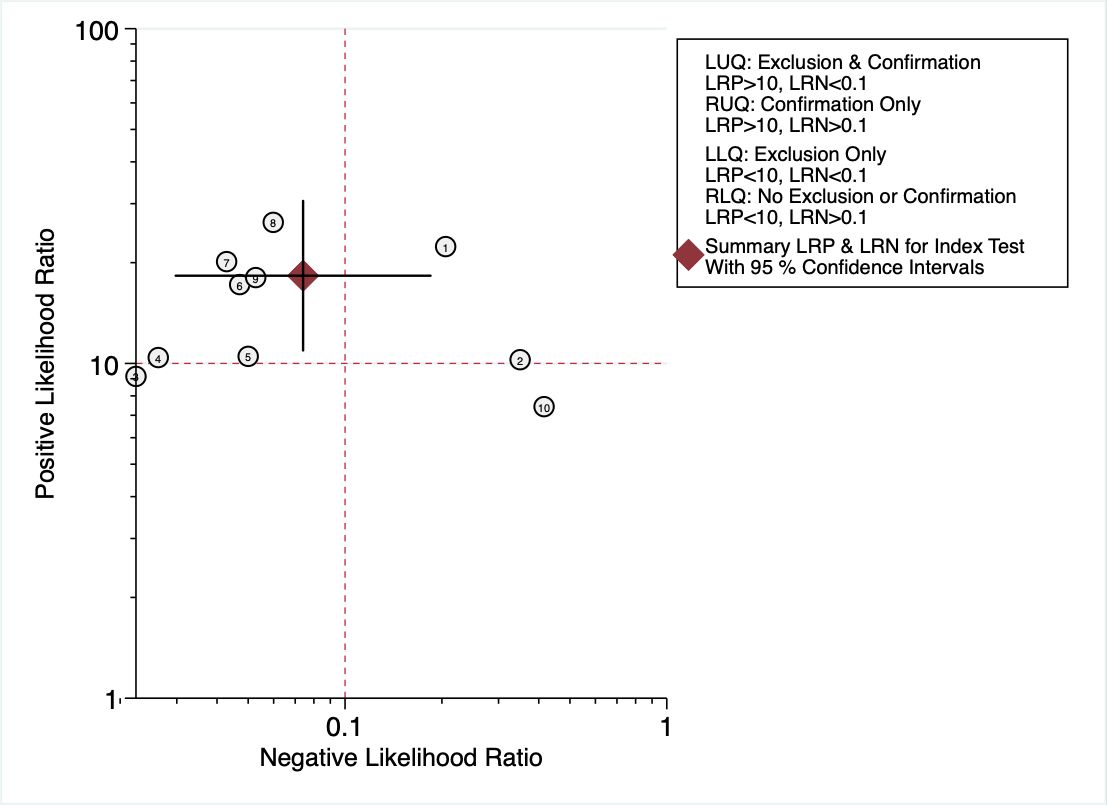

Supplement: Supplementary Figure 3 — Summary of positive likelihood ratio and negative likelihood ratio for the diagnosis of PJI. LLQ, left lower quadrant; LRN, likelihood ratio negative; LRP, likelihood ratio positive; LUQ, left upper quadrant; RLQ, right lower quadrant; RUQ, right upper quadrant. [file Image_3.tif]

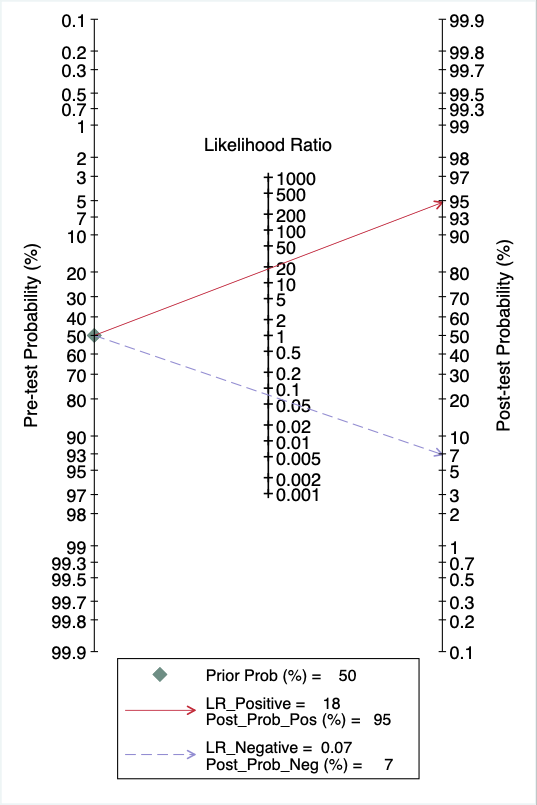

Supplement: Supplementary Figure 4 — Fagan nomogram of the mNGS for the diagnosis of PJI. [file Image_4.tif]
