# Supplementary material for: The Effectiveness of Metagenomic Next-Generation Sequencing in the Diagnosis of Prosthetic Joint Infection: A Systematic Review and Meta-Analysis
Source: Front Cell Infect Microbiol. 2022 Jun 10;12:875822. doi: 10.3389/fcimb.2022.875822 (PMC9226560; doi:10.3389/fcimb.2022.875822)
Supplement: Supplementary file 5 [file DataSheet_1.docx]

Supplementary Material

# Supplementary Data

## S1. Search strategies

### Searching in PubMed

((((((prosthetic joint infection) OR (periprosthetic joint infection)) OR (PJI)) OR (prosthesis-related infections)) OR (prosthesis infection)) OR (infection)) AND (((((((metagenomic sequencing) OR (mNGS)) OR (metagenomic next-generation sequencing)) OR (shotgun metagenomics)) OR (genomics)) OR (genetic diagnosis)) OR (sequencing))

### Searching in OVID

#1 (prosthetic joint infection or periprosthetic joint infection or PJI or prosthesis-related infections or prosthesis infection or infection).af.

#2 (metagenomic sequencing or mNGS or metagenomic next-generation sequencing or shotgun metagenomics or genomics or genetic diagnosis or sequencing).af.

#3 (#1 and #2)

## S2. Analysis of Diagnostic Threshold

# Supplementary Tables

**Table S1.** The Detailed Quality Information of the Included Studies.

| **Study** | **Thoendel 2018** | **Ivy 2019** | **Zhang 2019** | **Huang 2019** | **Cai 2020** | **Wang 2020** | **Huang 2020** | **Fang 2020** | **He 2021** | **Yu 2021** |
| --- | --- | --- | --- | --- | --- | --- | --- | --- | --- | --- |
| **Patient selection** |  |  |  |  |  |  |  |  |  |  |
| **Signaling question 1** | **Yes** | **Yes** | **Yes** | **Yes** | **Yes** | **Yes** | **Yes** | **Yes** | **Yes** | **Yes** |
| **Signaling question 2** | **Yes** | **Yes** | **Yes** | **Yes** | **Yes** | **Yes** | **Yes** | **Yes** | **Yes** | **Yes** |
| **Signaling question 3** | **Yes** | **Yes** | **Yes** | **Yes** | **Yes** | **Yes** | **Yes** | **Yes** | **Yes** | **Yes** |
| **Risk and bias** | **LR** | **LR** | **LR** | **LR** | **LR** | **LR** | **LR** | **LR** | **LR** | **LR** |
| **Applicability** | **LC** | **LC** | **LC** | **LC** | **LC** | **LC** | **LC** | **LC** | **LC** | **UC** |
| **Index test** |  |  |  |  |  |  |  |  |  |  |
| **Signaling question 1** | **Unclear** | **Unclear** | **Yes** | **Yes** | **Unclear** | **Unclear** | **Yes** | **Yes** | **Unclear** | **Unclear** |
| **Signaling question 2** | **Yes** | **Yes** | **Yes** | **Yes** | **Yes** | **Yes** | **Yes** | **Yes** | **Yes** | **Yes** |
| **Risk and bias** | **UR** | **UR** | **LR** | **UR** | **UR** | **UR** | **LR** | **LR** | **UR** | **LR** |
| **Applicability** | **LC** | **LC** | **LC** | **LC** | **UC** | **UC** | **LC** | **LC** | **LC** | **UC** |
| **Reference standard** |  |  |  |  |  |  |  |  |  |  |
| **Signaling question 1** | **Yes** | **Yes** | **Yes** | **Yes** | **Yes** | **Yes** | **Yes** | **Yes** | **Yes** | **Yes** |
| **Signaling question 2** | **Yes** | **Yes** | **Unclear** | **Yes** | **Yes** | **Unclear** | **Yes** | **Yes** | **Yes** | **Yes** |
| **Risk and bias** | **LR** | **LR** | **UR** | **LR** | **LR** | **UR** | **LR** | **LR** | **LR** | **LR** |
| **Applicability** | **LC** | **LC** | **LC** | **LC** | **LC** | **LC** | **LC** | **LC** | **LC** | **LC** |
| **Flow and timing** |  |  |  |  |  |  |  |  |  |  |
| **Signaling question 1** | **Yes** | **Yes** | **Yes** | **Yes** | **Yes** | **Yes** | **Yes** | **Yes** | **Yes** | **Yes** |
| **Signaling question 2** | **Yes** | **Yes** | **Yes** | **Yes** | **Yes** | **Yes** | **Yes** | **Yes** | **Yes** | **Yes** |
| **Signaling question 3** | **Yes** | **Yes** | **Yes** | **Yes** | **Yes** | **Yes** | **Yes** | **Yes** | **Yes** | **Yes** |
| **Risk and bias** | **LR** | **LR** | **LR** | **LR** | **LR** | **LR** | **LR** | **LR** | **LR** | **LR** |

Patient selection

Signaling question 1: Was a consecutive or random sample of patients enrolled?

Signaling question 2: Was a case-control design avoided?

Signaling question 3: Did the study avoid inappropriate exclusions?

Index test

Signaling question 1: Were the index test results interpreted without knowledge of the results of the reference standard?

Signaling question 2: If a threshold was used, was it prespecified?

Reference standard

Signaling question 1: Is the reference standard likely to correctly classify the target condition?

Signaling question 2: Were the reference standard results interpreted without knowledge of the results of the index test?

Flow and timing

Signaling question 1: Was there an appropriate interval between the index test and reference standard?

Signaling question 2: Did all patients receive the same reference standard?

Signaling question 3: Were all patients included in the analysis?

HC, high concern; HR, high risk; LC, low concern; LR, low risk; UC, unclear concern; UR, unclear risk.

**References**

Thoendel MJ, Jeraldo PR, Greenwood-Quaintance KE, Yao JZ, Chia N, Hanssen AD, et al. Identification of Prosthetic Joint Infection Pathogens Using a Shotgun Metagenomics Approach. Clin Infect Dis. 2018;67(9):1333-8.

Ivy MI, Thoendel MJ, Jeraldo PR, Greenwood-Quaintance KE, Hanssen AD, Abdel MP, et al. Direct Detection and Identification of Prosthetic Joint Infection Pathogens in Synovial Fluid by Metagenomic Shotgun Sequencing. J Clin Microbiol. 2018;56(9).

Zhang CJ, Fang XY, Huang ZD, Li WB, Zhang CF, Yang B, et al. Value of mNGS in sonication fluid for the diagnosis of periprosthetic joint infection. Arthroplasty. 2019;1(1):9.

Huang ZD, Zhang CJ, Li WB, Fang XY, Zhang CF, Yang B, et al. Detection of periprosthetic joint infection pathogens by metagenomic next⁃generation sequencing. Chin J Orthop. 2019;39:944-53.

Cai Y, Fang X, Chen Y, Huang Z, Zhang C, Li W, et al. Metagenomic next generation sequencing improves diagnosis of prosthetic joint infection by detecting the presence of bacteria in periprosthetic tissues. Int J Infect Dis. 2020;96:573-8.

Wang CX, Huang Z, Fang X, Li W, Yang B, Zhang W. Comparison of broad-range polymerase chain reaction and metagenomic next-generation sequencing for the diagnosis of prosthetic joint infection. Int J Infect Dis. 2020;95:8-12.

Huang Z, Li W, Lee GC, Fang X, Xing L, Yang B, et al. Metagenomic next-generation sequencing of synovial fluid demonstrates high accuracy in prosthetic joint infection diagnostics: mNGS for diagnosing PJI. Bone Joint Res. 2020;9(7):440-9.

Fang X, Cai Y, Shi T, Huang Z, Zhang C, Li W, et al. Detecting the presence of bacteria in low-volume preoperative aspirated synovial fluid by metagenomic next-generation sequencing. Int J Infect Dis. 2020;99:108-16.

He R, Wang Q, Wang J, Tang J, Shen H, Zhang X. Better choice of the type of specimen used for untargeted metagenomic sequencing in the diagnosis of periprosthetic joint infections. Bone Joint J. 2021;103-b(5):923-30.

Yu Y, Zhang SK, Lu ST, Tan J, Li Y, Xu JZ. Performance of metagenomic next⁃generation sequencing in diagnosis of prosthetic joint infection. Chin J Orthop. 2021;41(05):280-8.

# Supplementary Figures

Fig. S1. Univariable meta-regression.

Fig. S2. The Deeks’ funnel plot of the pooled DOR. DOR, diagnostic odds ratio; ESS, effective sample size.

Fig.S3. Summary of positive likelihood ratio and negative likelihood ratio for the diagnosis of PJI. LLQ, left lower quadrant; LRN, likelihood ratio negative; LRP, likelihood ratio positive; LUQ, left upper quadrant; RLQ, right lower quadrant; RUQ, right upper quadrant.

Fig.S4. Fagan nomogram of the mNGS for the diagnosis of PJI.
